# Supplementary material for: Content-rich biological network constructed by mining PubMed abstracts
Source: BMC Bioinformatics. 2004 Oct 8;5:147. doi: 10.1186/1471-2105-5-147 (PMC528731; doi:10.1186/1471-2105-5-147)
Supplement: Additional File 2 — The original results of the above study (non-essential files are deleted to keep the file size under the limit set by BMC bioinformatics). [file 1471-2105-5-147-S2.bz2 › chilibotAdditionalFile2/dip20/428ID7622448E10358/html/CCR1_CCL3.html]

 


 **CCR1** and **CCL3** 
  
Found 74 abstracts in PubMed, retrieved 20.  
 

 What does Google say? 
 PDF only 
| .edu only 

---

**Interactive relationship** (e.g. stimulation, inhibition, etc)

**Stimulatory relationship**- Eosinophils from these donors showed increased eosinophil  **CCR1**  expression and also underwent  **CCL3**  mediated chemotaxis and up regulation of CD11b.  Ref: 12794150 J Immunol, 2003
- Thus, IL 1 may enhance the local production of  **CCL3** , which may interact with  **CCR1**  expressed on hepatoma cells, in an autocrine and or paracrine manner.  Ref: 12651617 Am J Pathol, 2003
- CCL9, which acts through  **CCR1** , stimulated cytoplasmic motility and polarization in osteoclasts, identical to that previously observed in response to  **CCL3**  MIP 1alpha, which also acts through  **CCR1**  and is chemotactic for osteoclasts.  Ref: 12397598 J Cell Biochem, 2002
- Moreover,  **CCL3**  L1, but not  **CCL3** , is susceptible to cleavage by CD26, creating a truncated 2 form with enhanced affinity for  **CCR1**  and CCR5.  Ref: 12355456 Eur J Immunol, 2002
**Neutral relationship**- However, the remaining expression of  **CCR1**  and CCR5 correlated reciprocally with an ongoing unresponsiveness to the  **CCR1**  and CCR5 agonists  **CCL3**  macrophage inflammatory protein 1alpha MIP 1alpha, CCL4 MIP 1beta, and CCL5 regulated on activation, normal T expressed and secreted RANTES, all chemokines binding to these two receptors.  Ref: 12885942 J Leukoc Biol, 2003
- One such receptor is  **CCR1**  which is a receptor for CC chemokines, such as CCL5 RANTES and  **CCL3**  MIP 1alpha.  Ref: 12769747 Curr Pharm Des, 2003
- Potential Interaction between  **CCR1**  and Its Ligand,  **CCL3** , Induced by Endogenously Produced Interleukin 1 in Human Hepatomas.  Ref: 12651617 Am J Pathol, 2003

**Non-interactive relationship** (e.g. studied together, co-existance, homology, etc.)

- We also evaluated the expression of the  **CCR1**  ligand macrophage inflammatory protein 1alpha MIP 1alpha  **CCL3** .  Ref: 12541321 J Comp Neurol, 2003
- Moreover, the forskolin mediated increases in intracellular cAMP concentrations were inhibited by the ligands for  **CCR1** ,  **CCL3** , CCL4, and CCL5, suggesting that the expressed  **CCR1**  was functional.  Ref: 12651617 Am J Pathol, 2003
- To test the in vivo relevance of this, we used a murine melanoma system and knockout mice to investigate the function of the chemokine receptor CCR5 and its ligands, CCR  [ **CCR1** ]  ligand CCL 3  [ **CCL3** ]  and CCL5.  Ref: 12682253 J Immunol, 2003
- The expression of CXCR4 and  **CCR1**  and the migration to their ligands, SDF 1, and RANTES and MIP 1 alpha  [ **CCL3** ] , respectively, were also demonstrated in primary MM cells.  Ref: 12529679 Leukemia, 2003
- In contrast,  **CCL3** , a ligand of  **CCR1**  and CCR5, induced rapid uptake of FITC dextran by immature DCs but not mature DCs.  Ref: 12609829 Blood< MedlineTA>Blood, 2003
- Our studies showed that LD infection caused the upregulation of three beta chemokines macrophage inflammatory protein 1 alpha  [ **CCL3** ]  MIP 1alpha, MIP 1beta and RANTES regulated on activation normal T cell expressed and secreted, one alpha chemokine interleukin 8 IL 8 and the CC chemokine receptor 5 CCR5 but not  **CCR1** , as evident from reverse transcriptase polymerase chain reaction RT PCR analysis.  Ref: 12662300 Scand J Immunol, 2003
- In parallel,  **CCR1**  and CCR5 expression is down regulated and CXCL8, CCL2,  **CCL3**  and CCL5 production is up regulated.  Ref: 12548563 Eur J Immunol, 2003
- After 2 to 4 weeks, L. amazonensis infected mice had significantly delayed and depressed expression of inflammatory cytokines interleukin 12 IL 12, gamma interferon, IL 1alpha, IL 1beta, CC chemokines CC chemokine ligand 3  [ **CCL3** ]   **CCL3**  macrophage inflammatory protein 1alpha MIP 1alpha, CCL4 MIP 1beta, CCL5 RANTES, MIP 2, and chemokine receptors  **CCR1** , CCR2, CCR5 in foot tissues and draining lymph nodes compared to the expression in L. major infected controls.  Ref: 12874303 Infect Immun, 2003
- Although only a minority of the cell lines responded by calcium mobilization after agonist stimulation, a migratory response to the  **CCR1**  ligands RANTES and MIP 1 alpha  [ **CCL3** ]  was obtained in 5 6 and 4 6, respectively, of the cell lines tested.  Ref: 12529679 Leukemia, 2003
